# iRun: Horizontal and Vertical Shape of a Region-Based Graph Compression

**DOI:** 10.3390/s22249894

**Published:** 2022-12-15

**Authors:** Muhammad Umair, Young-Koo Lee

**Affiliations:** Department of Computer Science and Engineering, Kyung Hee University, Global Campus, Yongin-si 17104, Republic of Korea

**Keywords:** graph compression, graph computation algorithms, graph mining, adjacency matrix regions

## Abstract

Graph data are pervasive worldwide, e.g., social networks, citation networks, and web graphs. A real-world graph can be huge and requires heavy computational and storage resources for processing. Various graph compression techniques have been presented to accelerate the processing time and utilize memory efficiently. SOTA approaches decompose a graph into fixed-size submatrices and compress it by applying the existing graph compression algorithm. This approach is promising if the input graph is dense. Otherwise, an optimal graph compression ratio cannot be achieved. Graphs such as those used by social networks exhibit a power-law distribution. Thus, applying compression to the fixed-size block of a matrix could lead to the empty cell processing of that matrix. In this paper, we solve the problem of ordered matrix compression on a deep level, dividing the block into sub-blocks to achieve the best compression ratio. We observe that the ordered matrix compression ratio could be improved by adopting variable-shape regions, considering both horizontal- and vertical-shaped regions. In our empirical evaluation, the proposed approach achieved a 93.8% compression ratio on average, compared with existing SOTA graph compression techniques.

## 1. Introduction

The data generated by real-world applications such as social networks, web networks, citation networks, biological data, and many others can be modeled as graphs. The size of the dataset generated by these domains can easily reach the petabyte scale, which leads to multidimensional research issues, e.g., storage, mining, and processing challenges in graph compression, a prominent active research field in data mining. Graph compression pursues the minimum number of bits to describe the input graph [1,2,3,4,5]. Furthermore, the goal of graph compression is to compress the input graph as much as possible to minimize storage space. Most graph compression algorithms compress the graphs to reduce the storage space but increase the processing overhead to decompress them before performing various mining operations. It must be noted that some graph compression algorithms compress the graphs to some extent and also speed up the mining operations performed directly on the compressed graphs [6]. The research community has tried to compress graphs based on domain-specific criteria. Graph operations such as querying neighbors in social networks [7] have resulted in the development of the “Eulerian data structure” to answer both in-neighbor and out-neighbor queries in near-linear time. In triangle listing, joining operations between adjacency lists [8,9,10] have provided some of the compressed graph mining solutions detailed in the existing work, dividing the matrix into fixed-size row-wise blocks, and storing only non-empty blocks [11].

**Figure 1:**
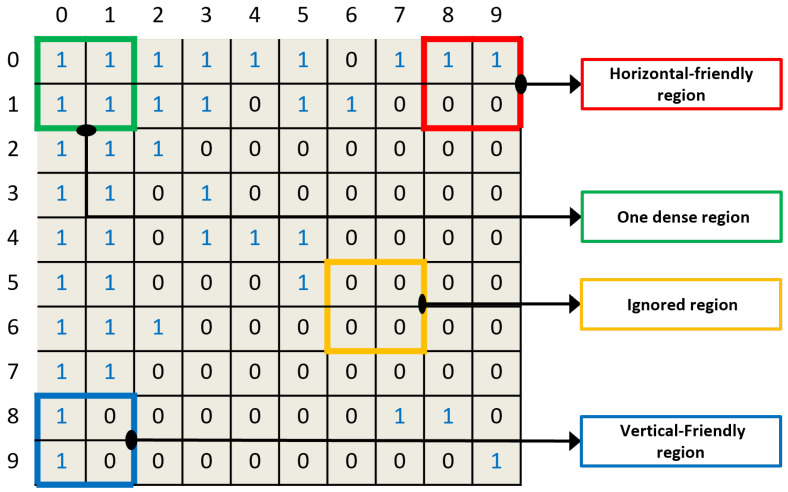
[Best viewed in color.] Compression-friendly regions of matrix *M* are shown in blue, green, and red color.

Real-world graphs have the power-law property, and thus consist of a few dense regions. The matrix representation of such graphs, as shown in Figure 1, consists of empty spaces (0 s) that may lead to a poor graph compression ratio. Furthermore, it can also cause high computation overheads by processing empty sub-matrices. In the prelude heuristic in Ref. [6], the matrix needs to be ordered in a compression-friendly manner. We choose the method given in Ref. [12] as the reordering technique used in this paper. The acquired matrix is an ordered matrix, subsequently employing reordering.

Moreover, the small number of dense blocks improves the compression ratio because the smaller blocks are better than the large number of sparse blocks. Therefore, the small number of dense blocks are suitable for compression. Secondly, this reduces the number of I/O accesses.

Therefore, the compression ratio of an ordered matrix can be improved by adopting differently shaped regions on a sub-sub-level rather than considering only fixed-sized regions. One can see four different possible regions of a matrix (e.g., horizontal-friendly region, one dense region, ignore region, and vertical-friendly region) in Figure 1. We divide the region into sub-sub-levels based on density and then compress each region (i.e., HH, VV) by applying various compression algorithms from the literature. However, none of the approaches are focused enough to achieve the optimal compression ratio and runtime. We address the issue of ordered matrix compression. On the other hand, to the best of our knowledge, very little research has been carried out previously to integrate the sub-sub-levels of the region compression algorithm to achieve better compression in the graph dataset.

Therefore, in this paper, we study the practical aspects of the proposed technique presented in Ref. [6], analyzing the proposed heuristic, implementation, and experimentation details. We name our technique the **h**orizontal and **v**ertical **r**egion (HVR), enabling efficient compression for the graph dataset. This paper is an extension of our previously published work. The key contributions of this paper are summarized as follows:

- We propose a lossless graph compression algorithm, iRun, which decomposes the ordered matrix into compression-friendly HVR, considering each block;
- We propose an architecture that allows compressing a graph using mixed graph compression algorithms at the block’s sub-sub-level to efficiently reduce storage space requirements;
- We compare our proposed technique with four existing bitmap compression algorithms and four encoding schemes for graph compression.
- Extensive experiments are carried out to validate the compactness and processing efficiency performance of the technique.

The rest of the paper is organized as follows. In Section 2, we describe the related work. In Section 3 we describe the proposed solution. We report extensive experimental results on the real-world graph dataset in Section 4. Finally, we conclude our work and provide future directions in Section 5.

## 2. Related Work

As graph size increases rapidly, massive graph processing is becoming a demand of the modern era. The domain of graph compression has been studied to speed up computation on massive graph data while using single consumer computers and distributed computing. One can find a composed survey on lossless graph compression and inverted index compression in Refs. [13,14], respectively. We divide the current graph compression techniques into two different types: graph-encoding techniques and bitmap compression.

### 2.1. Graph-Encoding Techniques

Adjacency lists and adjacency matrices typically represent graphs; these structures have been implemented in different file formats to store the graphs. This section is divided into the two main graph-encoding techniques.

#### 2.1.1. Encoding Adjacency List

The researchers in Ref. [15] suggested a compression technique for graphs on the web. The graph is compressed by representing adjacent nodes using gap encoding, instead of the original IDs, to achieve a space cast, because the original IDs (e.g., 32-bit word length) consume more space. In reference [16], the authors used the same idea of gap encoding.

#### 2.1.2. Encoding Adjacency Matrix

A wide range of existing studies represent graphs in the form of a matrix instead of a list [2,4,6,8,9,12,17,18,19]. Our work is closely related to Ace_Up [9], Ligra+ [10], and SlashBurn [12] in the context of improving the compression ratio. SlashBurn [12,17] and Ace_Up [9] are graph adjacency matrix-based systems. The matrices are divided into word-length-sized blocks. Real-world graphs lead to extremely large adjacency matrices that demand a large space in the system. Ligra+ [10] adopted different encoding techniques to compress the graphs.

Inside the adjacency matrix, SlashBurn [17] can co-cluster the non-zero elements more densely. It proposes recursively scrapping the highest-degree nodes first, and then finding the huge connected portion in the remaining graph. Then, it utilizes the traditional compression approach, gZip, to compress these blocks to reduce their memory footprint in the system. These techniques showed good performance, typically for graphs that exhibit a power-law distribution. On the other hand, SlashBurn has certain limitations. It is limited to undirected graphs and cannot be used directly to compress the directed graphs. Furthermore, gZip has a non-trivial compression overhead and is regarded as a heavy compression technique. Thus, compressed graphs cannot be processed instantly. Hence, they should be decompressed before any mining task is performed, which increases the computation overhead.

In Ace-Up [9], a hybrid approach is employed. Clustering is carried out first. Then, the adjacency matrix is represented by lists of encoded integers. The encoding includes the combination of 0s and 1s inside a block. For example, we consider the first (green) block of the matrix shown in Figure 1, a non-empty block in the row IDs 0 and 1. Its leftmost column ID is 0; the 2×2=4 binary elements (1111) can be encoded for an integer of 15. Thus, the integer pair 0 and 15 has been used to encode a block. The directory address of the given block could be 0, 1. A similar situation holds for other non-empty blocks. Combining the directory address and lists of integer pairs, such as the encoded list, produces the final structure of this technique. Compared with our proposed technique, we have the obvious benefit: the decoding overhead of the HVR encoding technique for original binary elements is trivial when compared with that of gZip or encoded list decompression. All the relevant compression techniques with iRun based on their feature comparison are given in Table 1.

The Ligra+ [10], a shared-memory graph-processing framework, is an extension of Blandford et al. [20]. They parallelized and integrated the compression and decoding technique to reduce the memory footprint, using k-bit and nibble codes, corresponding to 8-bit and 4-bit codes, respectively. With less memory, the compressed graph can be processed for sparse matrix–vector multiplication. In the processing of the matrix, the Nibble code is slower to decode due to the extra arithmetic required sequentially. In contrast, our proposed technique, iRun, is a partial parallel technique that performs best in a processing matrix, because the metadata (as described in Table 2) are sequential.

### 2.2. Bitmap Compression

**Figure 2:**
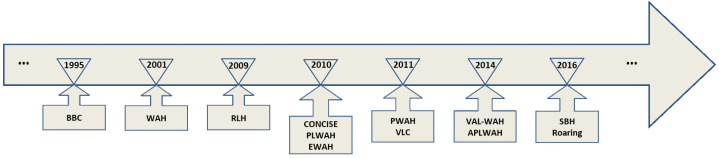
A brief history of compression algorithms.

Bitmap indexes have been widely considered as an efficient data structure for graph representation [22]. In Figure 2, we give a brief history of bitmap compression techniques [23,24,25,26,27].

Dealing with the storage requirements, a series of bitmap index-encoding algorithms have been proposed, such as BBC [28], WAH [29], CONCISE [30], EWAH [31], PWAH [32], and Roaring bitmap [33]. A detailed survey of these diverse bitmap compressions is given in Ref. [34].

In Ref. [31], Enhances Word Aligned Hybrid (EWAH) compression is the extended version of Ref. [29], which also uses only two types of words, as in WAH. The first type is a 32 bit verbatim word, and the second type is a marker word. EWAH bit maps begin with a marker word. The first bit indicates which clean word will follow: half of the 16 bits are used to store the number of clean words, and the remaining 15 bits are used to store the number of dirty words following the clean word. In the consecutive sequences of 216 identical clean words, the EWAH is less efficient than WAH because EWAH uses only 16 bits to store the number of clean words. In contrast, the HVR is more efficient in terms of compression ratio than EWAH because of the block’s novel horizontal and vertical partitioning. We studied different lengths of the blocks and showed the results in the experiments (see Section 4.2).

## 3. Proposed Solution

**Figure 3:**
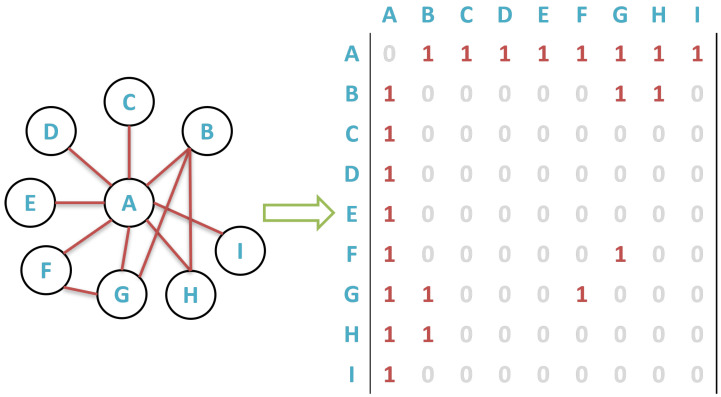
A graph *G*, and adjacency matrix *M*.

A graph is a collection of nodes with predetermined edges. Graphs can be of many sizes and shapes. An adjacency matrix is one method for displaying the nodes and edges in a graph. The nodes in a graph are assigned numbers from 1 to *N* to create the adjacency matrix. The N-by-N matrix’s elements (i,j) are then set to 1 if node *i* is connected to node *j*, and 0 otherwise. As a result, the adjacency matrix for undirected graphs is symmetric. There are multiple ways in which graph data can be represented in computer memory. In this paper, we define a graph *G* as an adjacency matrix *M*, see Figure 3, in which a region *r* could be the subpart of a matrix *M*, which will be divided into different shapes *S*. We represent a graph *G*, and the adjacency matrix *M*, with *n* nodes in our system (see Equation (1)).

(1) $$M_{ij}=\left\{\begin{array}{cc} 1 & \mathrm{if}\;\mathrm{an}\;\mathrm{edge}\;\mathrm{exists}\;\mathrm{between}\;\mathrm{nodes}\;i\;\mathrm{and}\;j\\ 0 & \mathrm{otherwise}. \end{array}\right.$$

This section provides the formal definition of the problem and describes the proposed methodology.

### 3.1. Problem Definition

**Figure 4:**
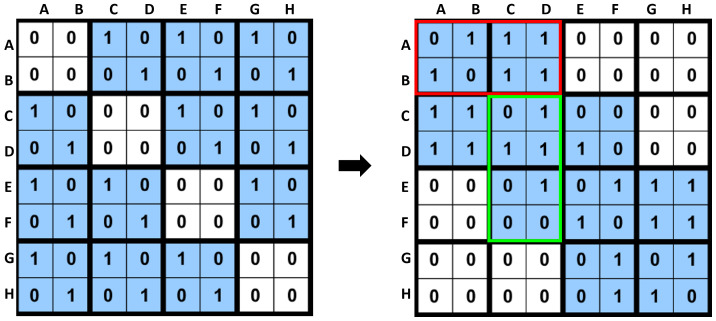
The importance of reordering for HVR. **Left**: the adjacency matrix is a part of the graph shown in Figure 3: A graph *G*, and the adjacency matrix *M* with the random ordering of nodes. **Right:** adjacency matrix of the same graph, but with a compression-friendly ordering.

We considered word-length-sized blocks of the adjacency matrix *M* of graph *G*. Each block was divided horizontally and vertically, strongly affecting the compression results of M. Thus, instead of using fixed-size blocks [6,9,12], each block was further divided into horizontal and vertical regions for a better compression ratio. In Figure 4, red and light green boxes show the compression-friendly horizontal and vertical regions, respectively. According to the heuristic proposed in Ref. [6], the matrix *M* needs to be ordered in a compression-friendly manner. The adjacency matrix of Figure 4 with a random ordering of nodes is on the left, whereas the adjacency matrix of the same graph with compression-friendly ordering is on the right. We assume that we cover all nonzero entries in the matrix with two-by-two blocks. The suitable matrix requires fewer blocks than the left matrix. In addition, each non-empty block in the suitable matrix is denser than the one in the left matrix, perhaps leading to better graph compression.

*Problem:* Given a graph *G* with adjacency matrix *M*, finding compression-friendly subsets of HVR implies a smaller number of bits such that the storage cost function, cost(M), is minimized.

The cost(M) is the required number of bits to represent the compressed matrix *M*. The more precise cost(M) uses the minimum number of bits to encode the matrix *M*. For adequate compression results, the matrix *M* is ordered using SlashBurn in a compression-friendly manner and obtains the matrix M_0 [12], as shown in Figure 4.

Different ordering techniques, such as [35,36,37], can impact compression, but reordering is out of the scope of this paper. The matrix considers word-length-sized blocks, and these blocks are decomposed into HVR. We formulated four Equations (2)–(5), to obtain the horizontal region, H_i, or the vertical region, V_i, of a block that can be divided into equal sizes of r×c.

(2) $$\left|r\right|=\frac{\left|br\right|}{2}$$

(3) |c|=|br|

For vertical blocks V_i, a block is divided vertically into equal-sized r×c regions such that

**Figure 5:**
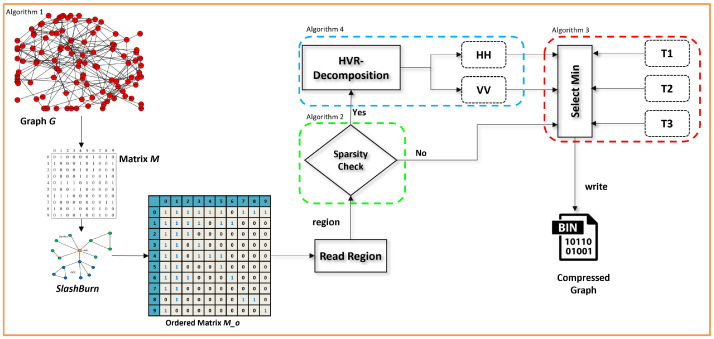
Detailed architecture of the proposed methodology. HH,VV are different shapes and T1,T2,T3 are the template algorithms.

(4) |r|=|bc|

(5) $$\left|c\right|=\frac{\left|bc\right|}{2}$$

where |r| represents the number of rows in H_i or V_i, |c| represents the number of columns in H_i or V_i, |br| represents the number of rows of a block, and |bc| represents the number of columns in a block. Finally, each horizontal and vertical region is encoded by the best suitable template (available algorithm) from the set of templates $\left\{T1,T2,T3,\ldots \right\}$. We considered the lowest compression cost for the horizontal (HH) or vertical (VV) region pairs in each sparse block. Figure 5 shows the detailed architecture of the proposed methodology. The architecture was divided into four algorithms.

### 3.2. Index Run Algorithm (iRun)

The iRun algorithm efficiently compresses the blocks in the horizontal and vertical regions in the form of key-value pairs. iRun is initiated by generating row vectors and column vectors for vertical and horizontal regions, respectively (for details, see Section 3.4). The generation of row vectors and column vectors helps in the efficient compression of sparse blocks, considering sequences of indexes in a different manner, e.g., row-wise and column-wise. Algorithm 1 shows the pseudo-code of the iRun algorithm.
**Algorithm 1:** iRun algorithm
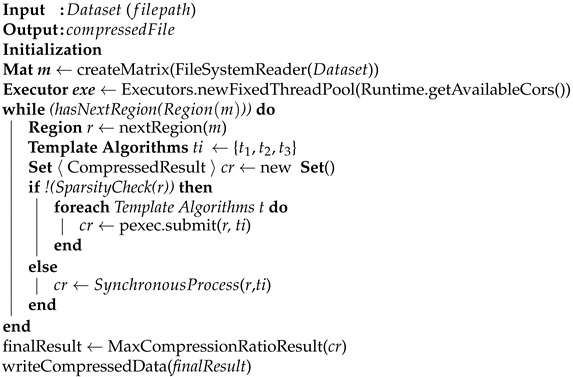


#### Sparsity Checking

Region *r* of block *b* may not always be sparse because of the power-law distribution [38], especially in the case of social networks, where some nodes have a vast number of degrees while others have fewer. Graph reordering and graph partitioning techniques also significantly affect the performance of graph compression techniques [9,17,39]. For example, in Figure 1, we can see the dense region highlighted in green. In order to reduce the processing overhead for such a region, we defined Algorithm 2. In contrast, we must find the best possible threshold value if the region r is sparse. We contemplated the data and determined the threshold value by defining Equation (6).

(6) $$thr=\frac{r\cdot size0}{3}$$

For example, if the region *r* has a size of 4×4, it has 16 indexes. If region *r* has one-third of non-empty indexes, it should be considered a dense and sparse region otherwise. We also tried to classify the regions as sparse and dense by considering half of the indexes of a block. The former method provides slightly improved performance as compared to the latter. We evaluated the difference between the threshold values in our experiment and provide an analysis in Section 4.
**Algorithm 2:** Sparsity Check algorithm
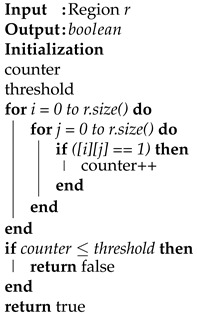


### 3.3. Parallel Process

The parallel process is the component of iRun that selects the best HVR template algorithm pair to obtain a high compression ratio in each block in parallel. When a region *r* has been confirmed as sparse by the previous step, the parallel process starts while taking that region *r* and the list of template algorithms as input. In order to achieve the best compression ratio, the parallel thread computes the best pair of shapes (horizontal or vertical). We defined Algorithm 3 for HVR decomposition with template algorithms (see Section 3.4), aiming to achieve the highest compression ratio of a pair (shape and algorithm). The best case was chosen among all pairs (see Algorithm 4).
**Algorithm 3:** HVR decomposition algorithm
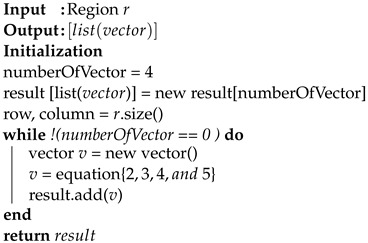


**Algorithm 4:** Synchronous Process algorithm

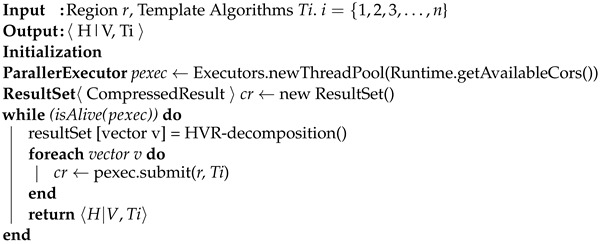



### 3.4. HVR Decomposition

**Figure 6:**
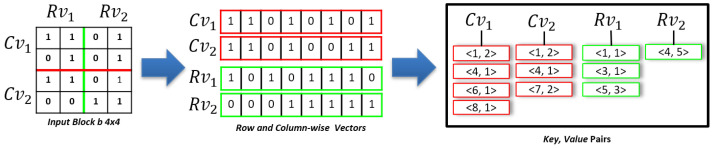
HVR-decomposition.

To obtain the horizontal and vertical region of an input block *b* of size 4×4, as shown in Figure 6, we divided block *b* into four vectors. In order to obtain the vectors, we formulated four Equations (2)–(5), as shown in Section 3.1. A row vector Rv_i is obtained by generating a row-wise sequence of indexes for a vertical region. In contrast, a column vector Cv_i is obtained by generating a column-wise sequence of indexes for a horizontal region, as shown in Figure 6. For each Rv_i and Cv_i, a set of key–value pairs is generated where the key represents an index offset of a sequence of 1s, and the value represents consecutive 1s in the sequence. For each block, horizontal or vertical region pairs with a small number of key–value pairs are chosen and stored on the disk.

The compressed data are stored in two files, in which one file contains the actual compressed data, while the second contains the metadata. Data can inexpensively be accessed directly from the file (see Table 2).

## 4. Experimental Setup and Evaluation

This section presents the experimental settings, including the experimental results and a comprehensive analysis of the proposed technique. We study the performance of iRun with four state-of-the-art encoding schemes and four SOTA bitmap compression algorithms. Furthermore, the decompression cost for the proposed iRun algorithm is also presented in this section.

All of the algorithms were developed in Java on a 64-bit Intel Core i5-4460 CPU 3.2 GHz, with four cores and three levels of caches running Windows 10. The experiments were conducted on various real-world datasets, as shown in Table 3, which provides the dataset statistics and specifications. Various compression algorithms are evaluated using memory and time complexity measures, but the focus is on evaluating the compression algorithms for compression ratios (cr). The compression ratio was calculated as follows:

(7) $$\;\mathrm{compressionRatio}\;\left(cr\right)=\frac{\;\mathrm{uncompressed}\;\mathrm{data}\;}{\;\mathrm{compressed}\;\mathrm{data}\;}$$

We evaluated the proposed technique in terms of three aspects: first, processing time in seconds; second, the space occupied on the disk by the compressed data; and finally, the compression ratio. This section shows the performance comparison of the proposed technique with the existing methods, considering the real-world graphs on a single consumer machine.

### 4.1. Processing Time Comparison

**Figure 7:**
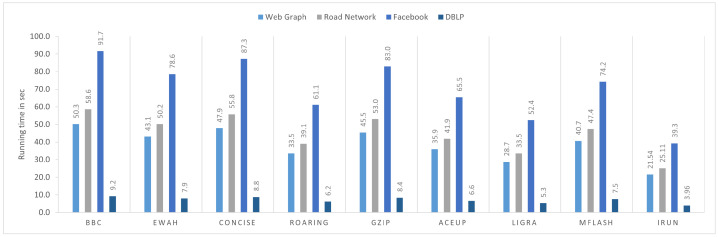
Processing time.

Real-world graphs, such as those from DBLP, Facebook, and WebGrph, except road networks, follow a power-law distribution and are hard to compress because they correspond to very sparse adjacency matrices. Figure 7 shows the processing time of each dataset compressed by different compression algorithms. In each dataset, iRun performs the best.

**Figure 8:**
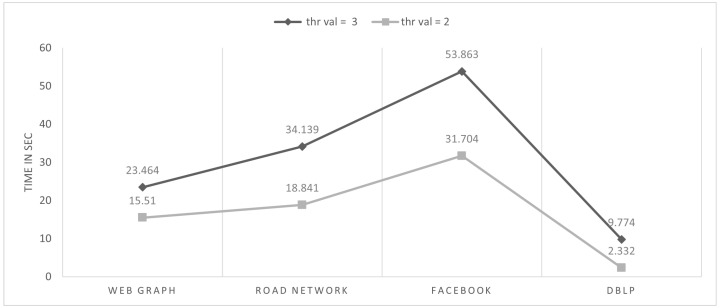
Processing time of each dataset choosing different threshold values.

To find the best optimum representation of the block and minimize the cost(M), we analyze the performance of Algorithm 2 by setting different threshold values. The results can be seen in Figure 8. Setting the threshold value to 3, we found that if the region *r* does not satisfy the condition, it is sparse. Moreover, the current region *r* is led toward the parallel process, which divides the region *r* into different shapes (horizontal and vertical) to find the best optimum for the compression ratio of the current region *r*.

### 4.2. Memory Complexity Comparison

iRun provides a better compression ratio than other graph compression techniques, such as Mflash, Ligra, AceUP, gZip, Roaring, CONCISE, EWAH, and BBC.

**Figure 9:**
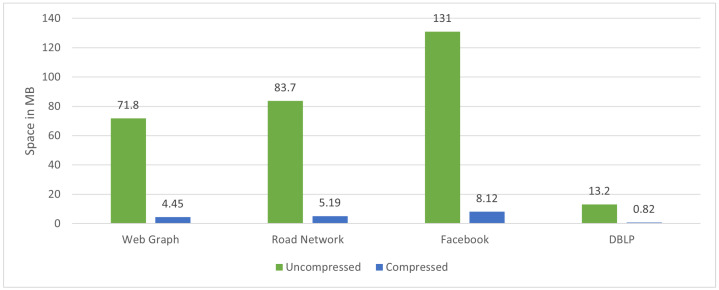
Uncompressed and compressed data on disk.

**Figure 10:**
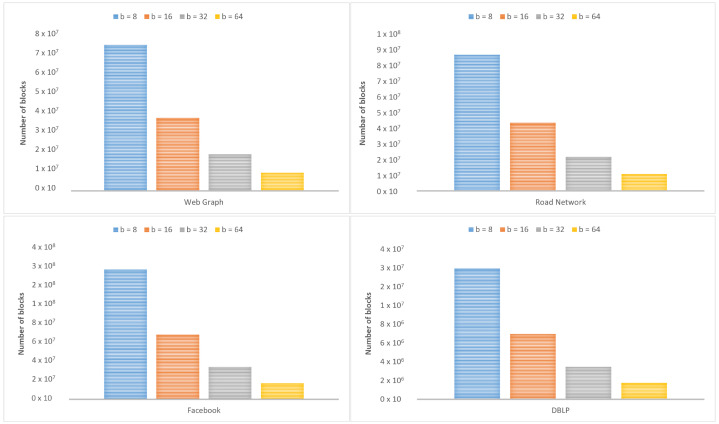
Number of non-empty blocks according to size of *b*.

**Figure 11:**
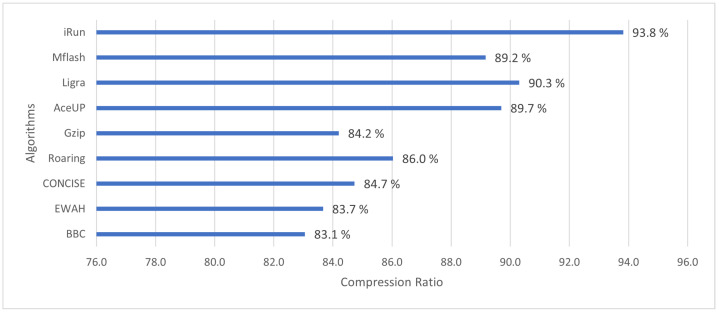
Compression ratio.

Figure 9 shows the standards of graph datasets before and after applying the iRun compression algorithm, showing the occupied space on the disk. In Figure 10, we look at the size of *b*, varying by 8, 16, 32, and 64, and divide graph adjacency matrices into b×b blocks. Parameter *b* will affect the number of non-empty blocks. Figure 11 shows the compression ratios for various compression algorithms. iRun outperforms the others by providing the lowest memory complexity because it utilizes the diversity of shapes and compression algorithms at horizontal and vertical levels of half-word-length size. For each HVR, row-wise and column-wise indices and the compression algorithm provide the highest compression ratio. We studied the size of the compressed matrix based on the cast function defined in Section 3.1. The effect of the parameter was analyzed. The performance of our proposed technique was studied in terms of memory.

Overall, a more significant *b* leads to fewer non-empty blocks. However, a larger *b*, for example, b=64, does not necessarily lead to a shorter processing time in graph computation because a larger *b* means more elements appearing in a block. Thus, we must spend more time making different shapes for *b*, in order to achieve a better compression ratio.

## 5. Conclusions

This paper proposes a lossless compression algorithm that compresses a graph at horizontal and vertical regions (HVR) in parallel for a variable length of block. We also propose the diversity of compression algorithms at the block level for the compression of a graph to obtain an efficient compression ratio. Row-wise and column-wise indices for HVR are generated and compressed using iRun in parallel. Our analysis shows that the small size of dense blocks leads to a much better compression ratio of 93.8% compared with the state-of-the-art compression algorithms. The iRun technique outperforms the existing compression algorithm on all real-world datasets, and more significantly on those that follow the power-law degree distribution. iRun preserves the actual state of the graph while maintaining the metadata. iRun shows promising results and can be investigated further in the future by elucidating its more silent properties. Currently, we are working on extending our technique to the distributed system.

## Figures and Tables

**Table 1:** A comparison of all the relevant compression techniques based on the properties of the input graph and the features of the algorithm.

System	Approach	Purpose	Computational	Distributed	Weighted	Lossless	Directed	Parallel
Ligra [21]	A parallel single- machine	Reducing the memory consumption	O(log3 M), M: sum of the sizes of the sets	✗	✓	✓	✓	✓
Ace Up [9]	Clustering, structural information of the graph	Directed graph compression	Sub-linear	✗	✗	✓	✓	✗
SlashBurn [12]	Reordering greedy hub selection	To reduce nonzero blocks in a resulting adjacency matrix	Iterated logarithmic	✓	✗	✓	✓	✗
HVR Graph Compression	Horizontal and vertical shaped compression	To achieve a higher compression ratio	Logarithmic	✗	✗	✓	✓	✓

**Table 2:** Meta file structure.

ID	Offset	Length	Shape	Template
0	0	5	$H_{1},H_{2}$	$T_{3},T_{3}$
1	5	15	$V_{1},V_{2}$	$T_{1}$
2	20	10	S	$T_{2}$
3	30	45	$H_{1},H_{2}$	$T_{2},T_{3}$

**Table 3:** Dataset statistics.

Real Graph Datasets	Type	# of Vertices	# of Edges	Size (MB)
Google Web Graph	Web pages network	875713	510503	71.8
Road Network	Road traffic network	196520	553321	83.7
Facebook	Social network	4847572	6899378	131
DBLP	Citation network	317080	104986	13.2

## References

[1] Liu Y., Safavi T., Dighe A., Koutra D. (2018). Graph Summarization Methods and Applications: A Survey. ACM Comput. Surv..

[2] Rasel M.K., Han Y., Kim J., Park K., Tu N.A., Lee Y.K. (2016). itri: Index-based triangle listing in massive graphs. Inf. Sci..

[3] Dhulipala L., Kabiljo I., Karrer B., Ottaviano G., Pupyrev S., Shalita A. Compressing graphs and indexes with recursive graph bisection. Proceedings of the 22nd ACM SIGKDD International Conference on Knowledge Discovery and Data Mining.

[4] Alam A., Umair M., Dolgorsuren B., Akhond M.R., Ali M.A., Qudus U., Lee Y.K. (2018). Distributed In-Memory Granularity-Based Time-Series Graph Compression.

[5] Dolgorsuren B., Khan K.U., Rasel M.K., Lee Y.K. (2019). StarZIP: Streaming graph compression technique for data archiving. IEEE Access.

[6] Umair M., Rasel M.K., Lee Y.K. (2017). BLOCK Formulation Technique for Compressed Graph Computation.

[7] Maserrat H., Pei J. Neighbor query friendly compression of social networks. Proceedings of the 16th ACM SIGKDD International Conference on Knowledge Discovery and Data Mining.

[8] Rasel M.K., Lee Y.K. Exploiting CPU parallelism for triangle listing using hybrid summarized bit batch vector. Proceedings of the 2016 International Conference on Big Data and Smart Computing (BigComp).

[9] Li G., Rao W., Jin Z. (2017). Efficient compression on real world directed graphs. Proceedings of the Asia-Pacific Web (APWeb) and Web-Age Information Management (WAIM) Joint Conference on Web and Big Data.

[10] Shun J., Dhulipala L., Blelloch G.E. Smaller and faster: Parallel processing of compressed graphs with Ligra+. Proceedings of the Data Compression Conference (DCC).

[11] Li G., Rao W. Compression-aware graph computation. Proceedings of the 2016 ACM International Joint Conference on Pervasive and Ubiquitous Computing: Adjunct.

[12] Lim Y., Kang U., Faloutsos C. (2014). Slashburn: Graph compression and mining beyond caveman communities. IEEE Trans. Knowl. Data Eng..

[13] Besta M., Hoefler T. (2018). Survey and taxonomy of lossless graph compression and space-efficient graph representations. arXiv.

[14] Pibiri G.E., Venturini R. (2020). Techniques for inverted index compression. ACM Comput. Surv. (CSUR).

[15] Boldi P., Vigna S. The webgraph framework I: Compression techniques. Proceedings of the 13th International Conference on World Wide Web.

[16] Boldi P., Santini M., Vigna S. (2009). Permuting web and social graphs. Internet Math..

[17] Kang U., Faloutsos C. Beyond’caveman communities’: Hubs and spokes for graph compression and mining. Proceedings of the 2011 IEEE 11th International Conference on Data Mining (ICDM).

[18] Kang U., Tong H., Sun J., Lin C.Y., Faloutsos C. Gbase: A scalable and general graph management system. Proceedings of the 17th ACM SIGKDD International Conference on Knowledge Discovery and Data Mining.

[19] Kang U., Tong H., Sun J., Lin C.Y., Faloutsos C. (2012). Gbase: An efficient analysis platform for large graphs. VLDB J.—Int. J. Very Large Data Bases.

[20] Blandford D.K., Blelloch G.E., Kash I.A. An Experimental Analysis of a Compact Graph Representation. Proceedings of the Sixth Workshop on Algorithm Engineering and Experiments and the First Workshop on Analytic Algorithmics and Combinatorics.

[21] Shun J., Blelloch G.E. (2013). Ligra: A lightweight graph processing framework for shared memory. Proc. Acm Sigplan Not..

[22] Chan C.Y., Ioannidis Y.E. Bitmap index design and evaluation. Proceedings of the 1998 ACM SIGMOD International Conference on Management of Data.

[23] Stabno M., Wrembel R. (2009). RLH: Bitmap compression technique based on run-length and Huffman encoding. Inf. Syst..

[24] Deliège F., Pedersen T.B. Position list word aligned hybrid: Optimizing space and performance for compressed bitmaps. Proceedings of the 13th International Conference on Extending Database Technology.

[25] Guzun G., Canahuate G., Chiu D., Sawin J. A tunable compression framework for bitmap indices. Proceedings of the 2014 IEEE 30th International Conference on Data Engineering.

[26] Corrales F., Chiu D., Sawin J. (2011). Variable length compression for bitmap indices. Proceedings of the International Conference on Database and Expert Systems Applications.

[27] Kim S., Lee J., Satti S.R., Moon B. (2016). SBH: Super byte-aligned hybrid bitmap compression. Inf. Syst..

[28] Antoshenkov G. Byte-aligned bitmap compression. Proceedings of the Data Compression Conference (DCC’95).

[29] Wu K., Otoo E.J., Shoshani A. (2006). Optimizing bitmap indices with efficient compression. ACM Trans. Database Syst. (TODS).

[30] Colantonio A., Di Pietro R. (2010). Concise: Compressed ‘n’composable integer set. Inf. Process. Lett..

[31] Lemire D., Kaser O., Aouiche K. (2010). Sorting improves word-aligned bitmap indexes. Data Knowl. Eng..

[32] van Schaik S.J., de Moor O. A memory efficient reachability data structure through bit vector compression. Proceedings of the 2011 ACM SIGMOD International Conference on Management of Data.

[33] Chambi S., Lemire D., Kaser O., Godin R. (2016). Better bitmap performance with roaring bitmaps. Softw. Pract. Exp..

[34] Chen Z., Wen Y., Cao J., Zheng W., Chang J., Wu Y., Ma G., Hakmaoui M., Peng G. (2015). A survey of bitmap index compression algorithms for big data. Tsinghua Sci. Technol..

[35] Barik R., Minutoli M., Halappanavar M., Tallent N.R., Kalyanaraman A. Vertex Reordering for Real-World Graphs and Applications: An Empirical Evaluation. Proceedings of the 2020 IEEE International Symposium on Workload Characterization (IISWC).

[36] Arai J., Shiokawa H., Yamamuro T., Onizuka M., Iwamura S. Rabbit order: Just-in-time parallel reordering for fast graph analysis. Proceedings of the 2016 IEEE International Parallel and Distributed Processing Symposium (IPDPS).

[37] Jacquelin M., Ng E.G., Peyton B.W. Fast and effective reordering of columns within supernodes using partition refinement. Proceedings of the 2018 Proceedings of the Seventh SIAM Workshop on Combinatorial Scientific Computing.

[38] Faloutsos M., Faloutsos P., Faloutsos C. (1999). On power-law relationships of the internet topology. ACM SIGCOMM Comput. Commun. Rev..

[39] Sun J., Vandierendonck H., Nikolopoulos D.S. Graphgrind: Addressing load imbalance of graph partitioning. Proceedings of the International Conference on Supercomputing.

